# Diversity within ovarian cancer subtypes and their tumor microenvironment

**DOI:** 10.3389/fonc.2026.1876327

**Published:** 2026-07-30

**Authors:** Lacey Winstone, Breanne Bevelander, Michael Dean Chamberlain

**Affiliations:** 1 Cancer Research Group, College of Medicine, University of Saskatchewan, Saskatoon, SK, Canada; 2 Department of Biochemistry, Microbiology & Immunology, College of Medicine, University of Saskatchewan, Saskatoon, SK, Canada; 3 Department of Oncology, College of Medicine, College of Medicine, University of Saskatchewan, Saskatoon, SK, Canada; 4 Department of Discovery and Translational Research, Saskatchewan Cancer Agency, Saskatoon, SK, Canada

**Keywords:** clear cell ovarian carcinomas (CCOC), endometrioid carcinomas, epithelial ovarian cancer (EOC), high-grade serous carcinoma (HGSC), low-grade serous carcinoma (LGSC), mucinous carcinomas, tumor microenvironment (TME)

## Abstract

Ovarian cancer is the deadliest gynecologic cancer in individuals assigned female at birth. Patients diagnosed with ovarian cancer experience poor survival rates due to frequent late-stage diagnosis and aggressive metastases. Ovarian cancer is often diagnosed at stages III or IV, where survival rates decline substantially. Ovarian cancer comprises several subtypes, each with distinct biological characteristics, clinical behaviors, and treatment responses, thereby complicating the identification of effective therapeutic targets. This review focuses primarily on the most prevalent subtype, high-grade serous carcinoma (HGSC) while also considering less common subtypes such as low-grade serous carcinoma, endometrioid carcinoma, clear cell and mucinous carcinomas. The tumor microenvironment (TME) plays a central role in tumor progression, metastasis, and immune evasion, thereby influencing therapeutic response and patient outcomes. Therefore, understanding the interactions between cancer cells and their microenvironment is essential for developing innovative therapeutic strategies. By identifying subtype-specific biomarkers and disease mechanisms, we can enhance diagnostic accuracy and develop tailored treatment approaches. A deeper understanding of ovarian cancer subtypes and their unique characteristics is vital for advancing patient care, driving innovations in early detection, and ultimately improving survival rates in this complex disease. This review emphasizes the potential for identifying novel biomarkers within the TME, which could facilitate earlier diagnosis and the development of targeted treatments for specific ovarian cancer subtypes, ultimately reducing mortality rates in this challenging cancer landscape.

## 1 Introduction

Ovarian cancer (OC) is a significant cause of cancer-related mortality among individuals assigned female at birth (AFAB) worldwide. The latest projections estimate OC incidences approximately 23,990 OC cases in Canada plus the USA, with a projected combined 14,730 deaths each year (1, 2). Overall, OC is highly metastatic, often resulting in the spread of cancerous cells within the abdomen, necessitating surgical intervention and chemotherapy (3). Individuals are typically diagnosed at 55–64 years of age (2), with 70–80% being diagnosed at stage III or IV (4). Delayed diagnosis contributes substantially to the high mortality rates. When detected at stage I, there is a 10-year survival rate of 73%; however, when diagnosed at stage IV, the overall 10-year survival rate is less than 5% (5). This sharp decline highlights the urgent need for early detection and more effective therapeutic strategies. Further investigation of the OC tumor microenvironment (TME) may identify novel therapeutic targets.

The TME is a complex mixture of cellular components, secreted factors from the blood, tumor, and omentum, plus extracellular matrix (ECM) components that surround and are in the tumor (6–9). The TME plays a crucial role by providing various signals and interactions that influence tumor behavior (10). The TME is essential for the survival and progression of the cancer, facilitating processes such as proliferation, migration, angiogenesis, immune response/evasion, and tumorigenesis (7, 8, 11–13). A comprehensive understanding of the TME is critical for advancing our knowledge of OC and for discovering novel biomarkers and patient treatment options.

### 1.1 Hereditary predisposition of ovarian cancer

Cancer is a multifactorial disease influenced by a combination of hereditary genetic mutations, environmental, and lifestyle factors that may contribute to epigenetic alterations. According to the Centers for Disease Control and Prevention (CDC), ~10-15% of all OC cases are due to hereditary mutations (14). Typically, *BRCA1/2* variants are the most inherited genetic alterations associated with OC, with a broad range of 5-83% of patients having at least one of these mutations (15–17). Other hereditary alterations include *PTEN* (18)*, STK11* (19), *TP53* (20, 21), and *KRAS* (22). Additionally, personal and family medical histories, such as a history of various cancers, polycystic ovarian syndrome (PCOS), endometriosis, and Lynch syndrome, can elevate the risk of developing OC (23–25). Although these factors increase OC risk, they do not inevitably lead to the disease.

### 1.2 Detection/treatments

Ovarian cancer is detected and diagnosed using a combination of imaging techniques, including ultrasound, computed tomography (CT) scans, X-rays, and magnetic resonance imaging (MRI). Those undergoing testing are typically at high risk of OC due to family history, referrals from gynecologic oncologists, or symptoms (26). Although the symptoms of OC often resemble other more benign conditions and may lead to testing and treatment for several other conditions before OC is considered, especially in individuals without a relevant family or genetic history (27, 28).

For patients diagnosed with OC, surgery remains the primary treatment option (29). Chemotherapy is typically administered following surgery and involves a platinum-taxane combination regimen (30–32). Unfortunately, only about two-thirds of OC patients initially respond to chemotherapy, and over time, ~80% of patients develop resistance (33–35). Poly (ADP-ribose) polymerase inhibitor (PARPi), including olaparib, niraparib, and rucaparib, have improved success rates as a first- or second-line therapy (36, 37), particularly in patients with mutations within BRCA1/2 or the homologous recombination DNA repair pathway (38). Despite advances in surgeries and treatments, challenges associated with late diagnosis, tumor heterogeneity, and therapeutic resistances continue to limit patient outcomes. Consequently, further investigation of the OC TME may reveal novel biomarkers and therapeutic targets.

### 1.3 Ovarian cancer types, subtypes and their progression

**Figure 1:**
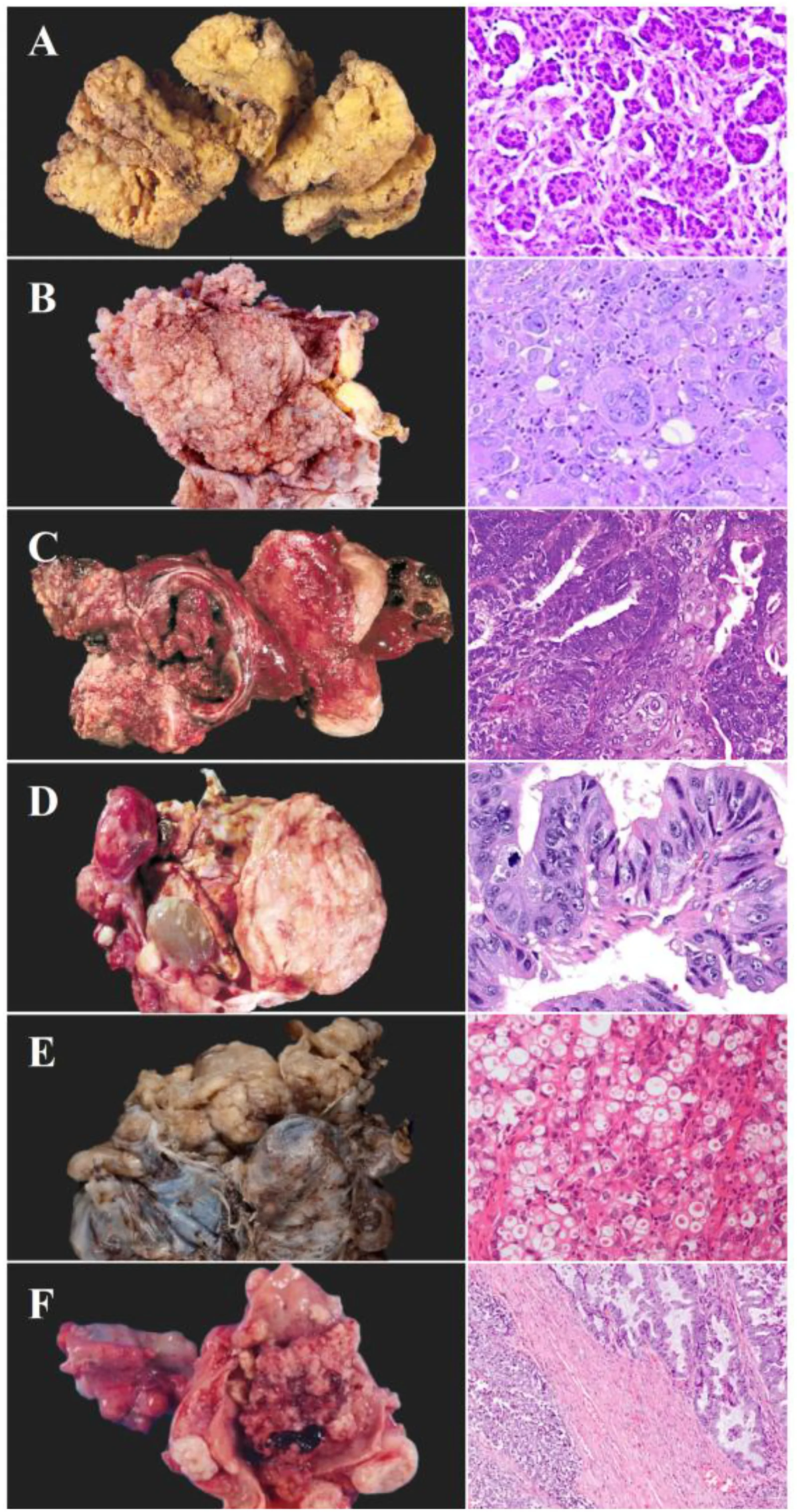
Gross and histological representation of ovarian cancer subtypes. Epithelial ovarian subtypes **(A–F)**. **(A)** Low-grade serous ovarian cancer. **(B)** High-grade serous ovarian cancer. **(C)** Endometrioid ovarian carcinoma. **(D)** Mucinous ovarian carcinoma. **(E)** Clear cell ovarian carcinoma. **(F)** Dedifferentiated ovarian carcinomas. Gross images created from (39–44). Cell images created with images from (43, 45–48).

**Figure 2:**
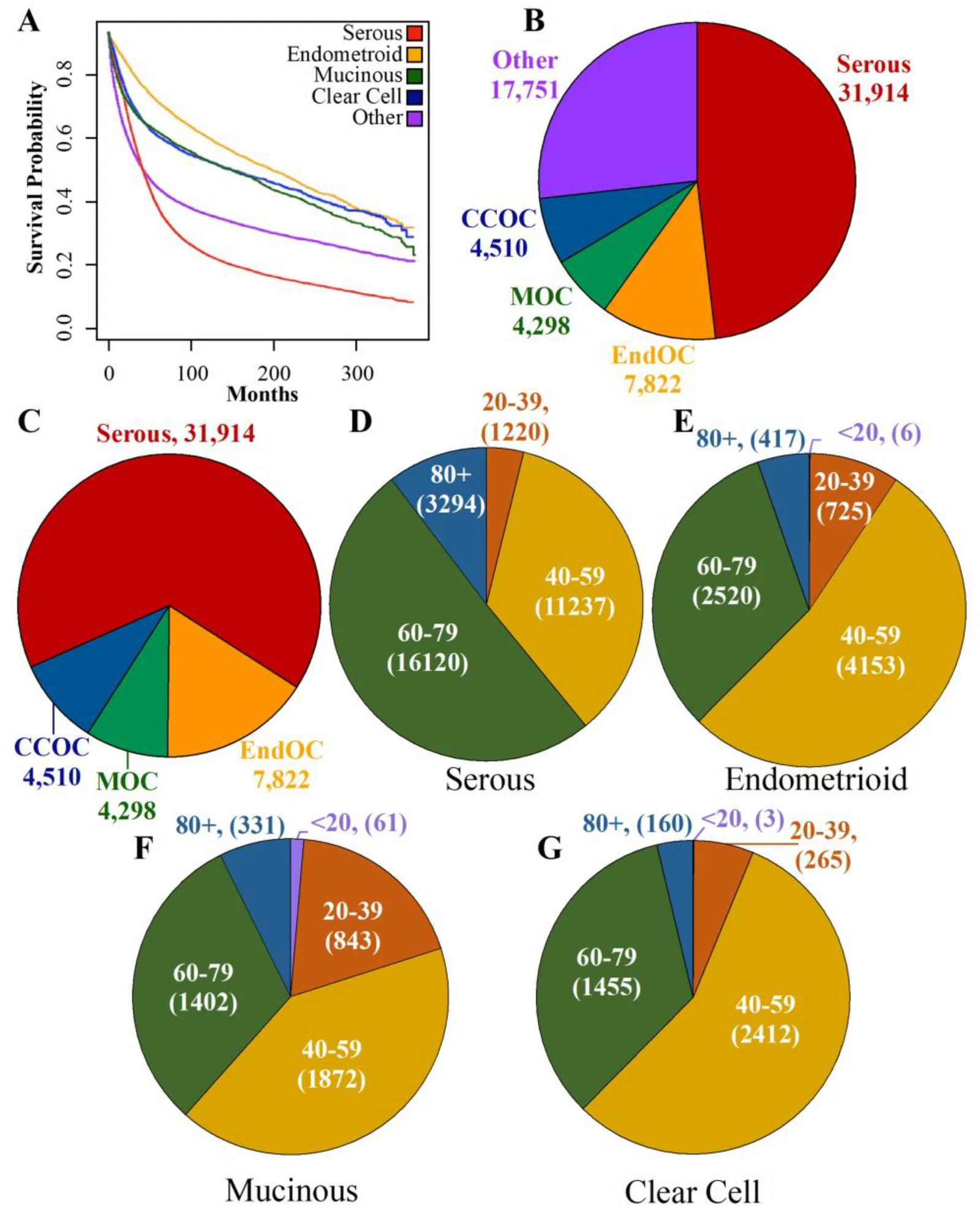
Survival and demographic distribution of ovarian cancer subtypes. **(A)** Overall survival probability for ovarian cancer (OC) patients (N=66295) from the SEER Database, separated by their designated subtypes. Endometrioid OC (EndOC), Mucinous OC (MOC), Clear Cell OC (CCOC). **(B)** Overall distribution of OC subtypes in the dataset. **(C)** Distribution of the epithelial OC subsets discussed in this paper, excluding the poorly defined “other” subtype. **(D–G)** Age distribution for serous, endometrioid, mucinous, and clear cell ovarian cancer, respectively. Data were extracted from the Surveillance, Epidemiology, and End Results (SEER) database for OC confirmed by positive histology from 1992-2022 (N=66295). Serous subtypes were not further subdivided into low-grade and high-grade, therefore both are listed under a single serous category. Dedifferentiated was not classified in this data set. Codes for the designated OC subtype were defined by (50, 51). Data collected from: (Surveillance Research Program, National Cancer Institute SEER*Stat Software Version 9.0.42.0., 2024) (52).

Ovarian cancer is a heterogenous disease comprised of several tumor types, each with distinct subtypes and classifications based on the origin site and histological archetype (**Figure 1**). Epithelial ovarian cancer (EOC), which accounts for 85-90% of all OC cases (49) (**Figure 2**). Other, rarer types include fallopian tube cancer, primary peritoneal cancer, germ cell OC, sex cord-stromal tumors, and borderline ovarian tumors. Each type may have numerous subtypes, and a single tumor can exhibit characteristics of multiple subtypes (53). Classification of the OC types and subtypes may vary across various studies. This paper will primarily focus on EOC, as it is the most common type of OC.

Epithelial OC tumors can be divided into five subtypes based on their morphology, origin, and unique landscape (54). These subtypes are serous carcinomas, endometrioid carcinomas, mucinous carcinomas, clear cell ovarian carcinomas (CCOC), and dedifferentiated ovarian carcinomas (DDOC). The distribution of these subtypes varies among different populations. For instance, serous carcinomas are the most prevalent EOC type among non-Hispanic White populations, while endometrioid and CCOC are more commonly found in Asian and Pacific Islander populations. In contrast, among non-Hispanic Black populations, sex cord-stromal tumors are the most frequently encountered (49). These population-specific differences highlight the importance of researching OC subtypes individually. This improvement may facilitate the development of treatments, and detection biomarkers that are tailored for specific subtypes, ensuring personalized care for all AFAB individuals at risk of OC.

Historically, the classification and origin of OC have been the subject of considerable debate. Initially, OC was thought to originate in the ovarian surface epithelium, the outer lining of the ovaries, and to be composed of cuboidal cells (55, 56), hence the term EOC. However, it is now recognized that serous carcinomas originate in the epithelium of the fallopian tubes, specifically the fimbriae, and that cancer cells are then released and grow on the ovaries (57–59). The complex pattern of the OC progression complicates staging, diagnosis, and therapeutic decision-making.

Serous carcinomas account for ~80% of all EOC cases (2, 60), and can be further classified into subtypes based on tumor grade: low-grade serous carcinoma (LGSC) and high-grade serous carcinoma (HGSC) (61). Serous tumors exhibit multiple growth patterns, often observed within a single lesion (62–64). Although both exhibit papillary structures, HGSC typically displays a more solid pattern with larger papillary formations than LGSC (62–65).

HGSC accounts for ~75% of all EOC cases (2) and is typically diagnosed at stage II or higher, with the median age of diagnosis being 57 years (66). HGSC is characterized by a near-universal *TP53* mutations and frequent homologous recombination deficiency, often driven by *BRCA1/2* alterations (54, 67) (**Table 1**). Histologically, HGSC cells exhibit significant nuclear atypia, large nuclei, and high mitotic rates (65). Patients often develop a resistance to chemotherapy over time, leading to poorer overall survival than LGSC (93, 94). As the most common subtype of OC and one of the most researched, HGSC still shows poor patient survival, highlighting the urgent need for earlier detection and improved treatment options for all subtypes of OC.

**Table 1:** Summary of genetic alterations and microenvironmental characteristics by ovarian cancer subtype.

OC subtype	Precent of EOC	Genetic alterations	Microenvironment	References
HGSC	*75%*	***TP53***, *BRCA1*, *BRCA2*, BRIP1, RAD51C, RAD51D, *STK11*	CAFs and TAMs enriched. Immunosuppressive. Often high levels of VEGF. High levels of CD8+ TILs.	(2, 54, 67–71)
LGSC	*5%*	***KRAS***, *NRAS, BRAF*, *USP9X, MACF1, ARID1A, NF2, DOT1L, ASH1L*	Lower levels of TAMs and PD-L1 than HGSC. Higher levels FAP+ CAFs and STING than HGSC.	(45, 60, 72–76)
Endometrioid OC	10-20%	**PTEN**, *CTNNB1* *(β-catenin), PIK3CA, ARID1A*	Lower level of TAMs, CAFs, T-cells (exhausted and total), PD-L1, and TGF-β than HGSC. Higher levels of CD44 and B7-H3 compared to other subtypes.	(54, 77–82)
Mucinous OC	*3-5%*	***KRAS***, TP53, *PIK3CA, CDKN2A, ERBB2, BRAF, SMAD4, SRC, CTNNB1* *(β-catenin)*, *FGR2, STK11*	Similar level of TAMs and PD-L1 as HGSC. Higher levels of CTLA-4 compared to HGSC. Higher levels of SMA, FAP+ CAFs, and Tregs compared to other subtypes.	(54, 82–85)
CCOC	*5-11%*	***ARID1A***, ***PIK3CA***, *TET2, TSC1, BRCA2, GNAS, SMAD4, MYC, NTRK1, KRAS*	Lower level of TAMs than HGSC. Similar levels of PD-L1 to HGSC. High levels of MET and phospho-PRAS40 proteins than HGSC.	(54, 79, 80, 82, 86–89)
DDOC	2%	**SWI/SNF** ***(BRG1 or IN11)***, ARID1A/ARID1B, SMARCA4, SMARCB1	Mostly unknown.	(90–92)

Ovarian cancer (OC) subtypes with alteration and microenvironment features from the literature. Genetic alterations in **bold** indicate the most frequent for that subtype. Microenvironment features are compared to HGSC or other subtypes. Increased levels, decreased levels, and similar levels in comparison are indicated with green, red, and blue, respectively. EOC, epithelial ovarian cancer. HGSC, high-grade serous ovarian cancer. LGSC, low-grade serous ovarian cancer. CCOC, clear cell ovarian carcinoma. DDOC, dedifferentiated ovarian carcinoma. CAFs, cancer associated fibroblasts. TAMs, tumor associated macrophages. VEGF, vascular endothelial growth factor. TILs, tumor-infiltrating lymphocytes. Tregs, regulatory T-cells. FAP, fibroblast activation protein. MET, mesenchymal-epithelial transition.

LGSC typically affects younger AFAB individuals in their 40s (95), with most diagnoses occurring at stage III (96). This subtype represents ~5% of EOC (60). Mutations in *KRAS*, *NRAS*, and *BRAF* genes are frequently observed in LGSC patients (72–74) (**Table 1**). These genes play roles in the MAPK pathway, which influences cellular growth, proliferation, apoptosis, cellular differentiation, and stress responses (97). Other mutations have been identified (in order of reducing frequency) in *USP9X, MACF1*, *ARID1A, NF2*, *DOT1L*, and *ASH1L* (75) (**Table 1**). LGSC cells are cuboidal with mild nuclear atypia, and a low mitotic rate (64). This subtype is associated with chemoresistance (98), which combined with diagnoses at later stages (96), results in poor survival rates for patients (99). Due to its rarity, survival estimates are not well defined; however, LGSC generally has a better prognosis than HGSC (93).

Endometrioid ovarian carcinomas account for ~10-20% of all EOC cases (54, 77) and are typically diagnosed around the age of 55 (100). Although, these carcinomas are more likely than the other subtypes to be diagnosed at younger ages and earlier stages, particularly in Asian/Pacific Islanders and Chinese populations (101, 102). Individuals with endometriosis face more than double the risk of developing this type of OC compared to those without the condition (25, 103–106). Additional risk factors include delayed menopause, family history of breast cancer (103), and low PTEN levels, which can disrupt the normal PI3K/AKT signaling pathway (78) (**Table 1**). This subtype originates in the ovarian epithelial cells and can spread through the abdomen and pelvis (107). These cells exhibit prominent nucleoli with round nuclei, with some atypia, resembling endometrial cells (42). The tumors can also have papillary structures but tend to look more cystic and solid (100). Due to earlier detection, patients with endometrioid OC generally have better prognoses (108). This highlights the importance of early detection in improving patient outcomes.

Mucinous ovarian carcinomas account for ~3-5% of all EOC cases (83) and typically affects individuals around ≤40 years of age (109). Smoking is the primary risk factor associated with this type of OC (110). These tumors are thought to originate on the surface of the ovaries and contain mucus-producing cells (84, 111). The mucus produced consists of glycoproteins known as mucins, which contribute to increased chemoresistance and tumor aggressiveness (84). The primary site of metastasis for mucinous OC is the gastrointestinal tract (112), which then spreads to other parts of the body. Mutations in the *ras* gene, particularly at codon 12 and, less frequently, at 13, are commonly observed in these tumors (84). The *KRAS* gene is also frequently mutated in this subtype (54) (**Table 1**), often found in benign, intermediate, and malignant areas of mucinous ovarian carcinomas; therefore, researchers are investigating these mutations as potential early indicators of the disease. Tumors are typically large and cystic with smooth external surfaces and may show papillary architecture (44, 113). Patients with mucinous OC generally have a lower survival (114).

Clear cell OCs account for ~5-11% of EOC cases and primarily affect individuals AFAB around 55 and are more frequently found in Asian populations (54, 86), specifically in individuals of Japanese descent (115). Under the microscope, CCOC cells appear transparent due to abundant cytoplasmic glycogen (116). Tumors can reach large sizes, and frequently have small papillary structures, cystic formations, and Hobnail cells (117). The origin of CCOC is not well understood, but it is hypothesized that, given its many similarities to HGSC, CCOC may arise and metastasize like HGSC. It may also arise from the peritoneum, which is often affected by endometriosis (105, 118, 119), with endometriosis being associated with approximately 35% of CCOC diagnoses (54). The CCOC shares morphological and molecular features with renal clear cell carcinomas (RCCC); therefore, treatments for RCCC may represent potential therapeutic avenues for CCOC such as the mTOR pathway and genes related to angiogenesis (54). Notably, mutations or loss of the *ARID1A* gene (87) and mutations in *PIK3CA* (88) are observed within CCOC (119) (**Table 1**). Patients with CCOC often exhibit resistance to drug therapies and have lower survival rates (120).

Dedifferentiated OC is the rarest EOC, accounting for only ~2% of cases, and is therefore the most under-researched and least understood (90). This subtype primarily affects individuals around 55 years old; however, it is more common in those under the age of 50 compared to some other subtypes (90). The growth pattern of these cells is irregular and is associated with deficiencies in the SWI/SNF complex (91) (**Table 1**). These cells tend to be metastatic, abnormally shaped, and poorly differentiated (121), which contributes to their aggressive nature. Tumors can vary in size and growth patterns, with high necrosis (122). As a result, patients often exhibit high resistance to chemotherapy and have a poor prognosis (123). The hypothesis regarding the origin of these cells suggests that they may have evolved from ovarian or endometrial cells that became differentiated due to mutations (48).

Epithelial OC is the most common type of OC. Even so, there are also rarer types, including germ cell and sex cord-stromal OC (124). Germ cell OC, accounting for ~2.6% of OC cases (125), arises from ovarian germ cells and can be categorized into several subtypes: dysgerminoma, teratoma, endodermal sinus tumor, embryonal carcinoma, choriocarcinoma, malignant struma ovarii, and mixed (126). On the other hand, sex-cord stromal ovarian tumors represent about 5% of all OC cases and are primarily found in young AFAB aged 15-24 (127, 128). This young age range is likely influenced by hormonal changes during development (129).

Overall, OC is relatively under-researched compared to other similarly deadly cancers, especially when regarding the rarer subtypes of OC. This lack of research has led to limited knowledge of variations within and between these subtypes, particularly in non-epithelial serous OC. To enhance our understanding of how these cancers behave, it is crucial to conduct research that explores variations in pathology and histology, identifies mutations that serve as biomarkers, and examines the resistance of different subtypes to specific therapies. Since OC is nearly impossible to self-detect and is often misdiagnosed due to its common symptoms, it is essential to explore early detection strategies to enable earlier diagnosis and improve overall survival rates. Unlike breast cancer, no widely implemented population screening programs exist for OC, which would provide a beneficial alternative as a frequent, non-invasive test to diagnose and potentially identify the specific subtype of OC.

## 2 Tumor microenvironment of ovarian cancer

**Figure 3:**
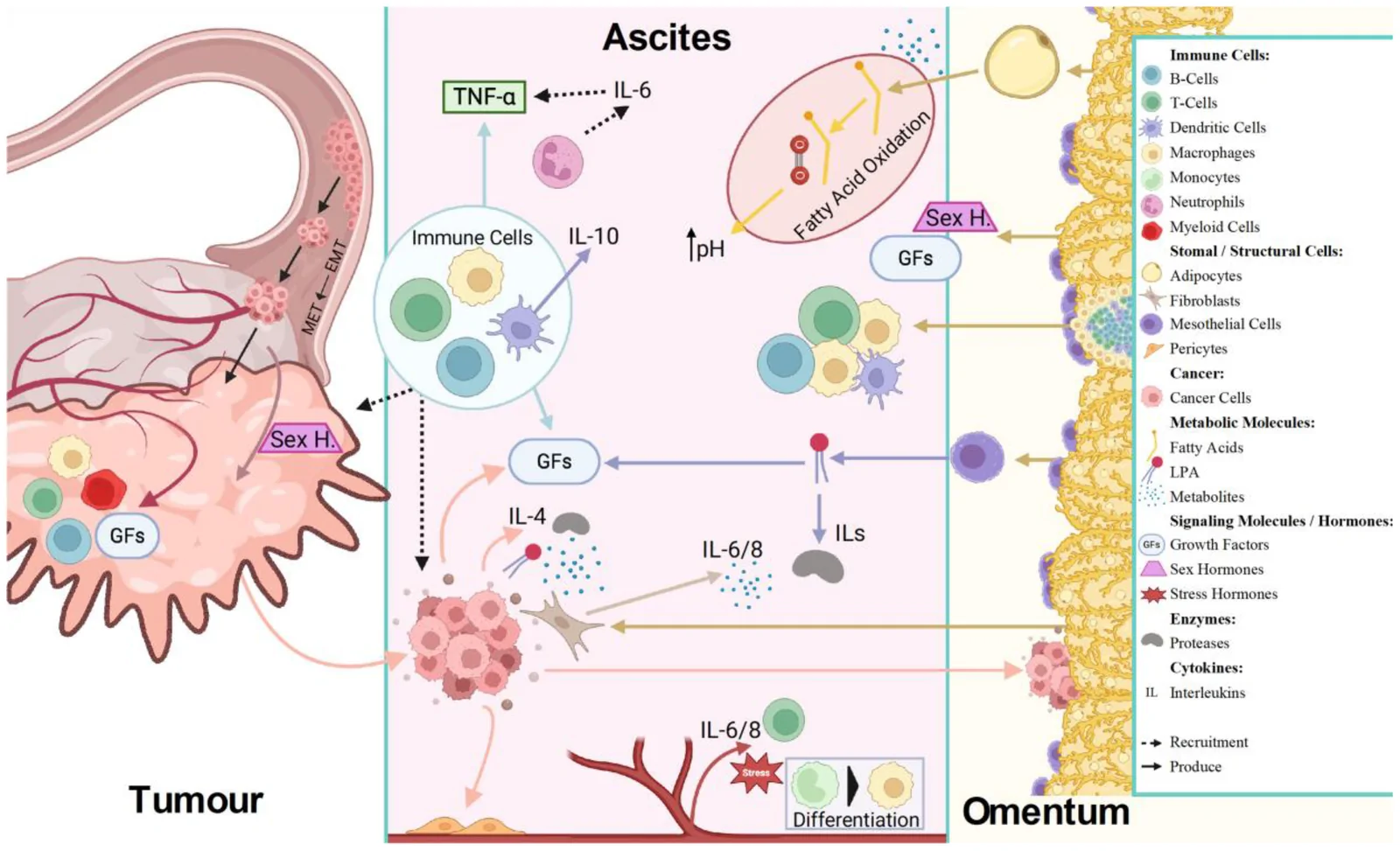
Generalized overview of the serous ovarian cancer tumor microenvironment. High-grade serous carcinoma (HGSC) is thought to originate from the fallopian tubes. Through metastatic progression, tumor cells may undergo epithelial-to-mesenchymal transition (EMT), then mesenchymal-to-epithelial transition (MET), the tumor establishes in the ovary. Ovarian cancer can also metastasize to the omentum. This schematic illustrates the interactions of cells, metabolites, hormones, enzymes, and cytokines from the ovary, tumor, blood systems, ascites, or the omentum. “Milky spots” are pockets of immune cells within the omentum. GFs, growth factors. SHs, sex hormones. LPA, lysophosphatidic acid. IL, interleukin. Solid arrows indicate cells/molecules/hormones produced from the indicated cell or organ. Dotted arrows indicate materials recruited to cells or molecules.

The tumor microenvironment (TME) is a complex environment that surrounds and infiltrates tumors, typically supporting tumor development. The TME consists of various components, including various cells (fibroblasts, T-cells, macrophages, dendritic cells, neutrophils, pericytes, endothelial cells, etc.), secreted factors (cytokines, proteases, metabolites, growth factors (GFs)), and ECM components (130, 131) (**Table 1**). The OC TME shares many characteristics with the microenvironment of other solid tumors (132). Nevertheless, given that HGSC tumors have now been shown to originate from the fallopian tubes (55, 56, 58, 59), and that the ovaries and fallopian tubes are exposed to the abdominal cavity, the TME of OC could be considered to have both a solid and a liquid compartment (133) (**Figure 3**). The liquid component of the OC TME consists primarily of peritoneal fluid of the abdominal cavity. An abnormal buildup of peritoneal fluid is termed ascites (134), and it is vital to differentiate between the solid and liquid phases of the OC TME when conducting research. However, many studies do not make this distinction and primarily focus on the solid TME. This review examines the different components of the OC, highlighting the contributions of both the solid and liquid compartments to OC development. We will break down the TME discussion into three parts: cellular components, ECM, and secreted factors.

### 2.1 The cellular components

As HGSC cells from the serous tubal intraepithelial carcinoma (STIC) in the fallopian tubes become more mesenchymal-like, they slough off the fallopian tube and establish themselves on the ovaries, where they return to a more epithelial-like state (135). This process occurs because of epithelial-to-mesenchymal transition (EMT) and mesenchymal-to-epithelial transition (MET), aiding in the malignant progression of the OC (135). The cells can also start proliferating on other tissues of the pelvic region, such as the omentum (136, 137), the peritoneum, a membrane lining the abdominal cavity that connects the abdominal organs, and the bladder (138). This liquid nature of the OC TME may facilitate early migration of cancer cells to secondary locations, unlike most cancers, causing OC to be highly aggressive and have high rates of relapse.

Single-cell sequencing studies comparing normal, and tumor associated tissues, revealed a general trend in how the tumor’s cellular microenvironment changes. These analyses include fallopian tube, ovarian, omental, and peritoneal fluid samples, although cellular composition varies according to subtype and stage. In the fallopian tubes of those with OC, there is an increased abundance of T-cells, fibroblasts, and epithelial cells, as well as some B-cells, endothelial cells, mast cells, myeloid cells, and pericytes (139, 140). Comparatively, tumors growing on the ovaries have increased counts of T-cells, myeloid cells, epithelial cells (the malignant cells), and B-cells, and a decrease in pericytes and fibroblasts compared to the normal ovary tissue (140). Ascites, on its own, is primarily composed of fibroblasts and epithelial cells, whereas the non-cancerous peritoneal fluids have higher levels of myeloid and B-cells in comparison (140, 141). Lastly, tumors on the omentum have higher levels of T-cells, mast cells, epithelial cells, and B-cells, with reduced fibroblasts and endothelial cells compared to normal omental tissue (140). These differences are consistent with progressive remodeling of the TME across primary and metastatic sites.

Ovarian cancer cells of epithelial morphology can comprise between 17-77.5% of tumor bulk (142, 143). Studies of the clonal population of the epithelial cells indicate that most tumors have multiple subpopulations of cancer cells within the tumor and that high levels of clonal expansion are correlated to poor prognosis (144). The immune cells are generally more numerous in the tumors with both a high fraction of myeloid and lymphoid cells. T- and B-cells contribute to anti-tumor immunity through recognition of tumor associated antigens and modulation of immune responses (145). As a result, higher levels of T-cells are associated with better EOC patient survival (146); however, B-cells show no impact on patient survival (147). Even so, as the tumor progresses, more of the T-cells become either exhausted or regulatory T-cells that dampen the immune response (148). A similar process occurs with B-cells (149). In OC, there is a reduction in the proportion of fibroblasts in the tumor (140); although, there is no evidence of fibroblasts reducing in numbers in tumors, this is likely due to the increase of epithelial cells (cancerous cells) changing the cellular ratios. The fibroblasts are also transformed into a cancer associated subtype; cancer-associated fibroblasts (CAFs) (150).

The fibroblasts/CAFs play countless roles in tumor development by secreting factors that can aid in growth, proliferation, and invasion of the tumor; as well as immune suppression (151–154). In OCs, high levels of CAFs expressing fibroblast activation protein (FAP) have been associated with higher invasion and chemoresistance as these CAFs showed higher expression of markers for migration, angiogenesis, immune response, and ECM remodeling (155). There is also a significant reduction in the number of pericytes in tumors which typically leads to instability of blood vessels (156). However, endothelial cell levels are very similar in normal and OC tissues (140), and therefore, blood vessel formation is relatively similar, but the blood vessels are more likely to be leaky due to pericyte loss (157). This is typical for blood vessel development within tumors (158).

Characterizing CAFs has revealed, depending on the marker panels used, four to eight distinct subclasses that could serve as diagnostic markers for OC (68, 159, 160). The CAFs are known to play pivotal roles in ECM production plus secretion of growth factors and immune modulating compounds (161). When normal fibroblasts were transformed *in vitro* into CAFs by OC cell lines (SKOV3, Endometroid, or Kuramochi, HGSC) conditioned media, the CAFs had increased secretion of Collagen, type 1, as well as, MMP-3, TIMP-1, VEGF, OPG, IGFBP-2, MCP-1, IL-8, and IL-6 (162). Interestingly the profiles of secreted factors by the CAFs were slightly different depending on the OC cell line used suggesting that different CAFs phenotypes are in some part driven by the cancer cells (162). Others have found similar results where CAFs express factors that drive OC progression. CRMP2 is secreted by CAFs and drives SKOV3 proliferation and increased tumor formation in mice (163). CAFs from OC also secrete FGF-1 that promotes cancer cell proliferation (164). The multifaceted roles of CAFs in tumorigenesis suggest their potential as therapeutic targets. Strategies to inhibit CAFs’ contributions to tumorigenic pathways could be pivotal in obstructing tumor survival pathways or mitigating adverse downstream effects like chemoresistance or immune modulation (165). Research investigating the effects of chemotherapy on the TME has illustrated how CAFs may enhance chemoresistance through the secretion of cytokines (150) and ECM-remodeling enzymes (160). And they also play a role in other mechanisms of tumor development, such as angiogenesis, metabolism, and immune suppression (166–168). As mentioned above there are different subclassifications of CAFs in OC patients and it has been shown by various studies that specific CAFs signatures are related to poor patient prognosis (155, 169, 170). This underscores the necessity for innovative approaches in TME-targeted therapies (171). Spatial sequencing has also shown the relevance of CAFs showing that a subset of patients with higher levels PDPN-positive CAFs had earlier relapse (159). These PDPN-positive CAFs also associated with abnormal tertiary lymphoid structures that did not have proper germinal centers and that where enriched with plasma cells (159). There have been nine clinical trials testing bortezomib, which targets proteasome activity and can affect plasma cells survival. However, all these trials closed either without reporting findings or with poor results suggesting that although plasma cells may have a role in OC tumor development (172) more research needs to be done to identify patients that could benefit from the targeting of plasma cells. All these studies point towards CAFs having a significant role in both driving the proliferation of OC and modulating the immune microenvironment during tumor development. A well-defined set of biomarkers to identify CAF subtypes as well as a better understanding of the factors that influence the development of the different subtype of CAFs in OC is an important area of future research. For example, recent studies of metastatic OC to the omentum suggest that some CAFs have origins as mesothelial cells and was driven by the upregulation of the RUNX1 transcription factor (173). Interestingly, proteomics analysis of HGSC and LGSC showed that LGSC had an enrichment of CAF markers (FAP, COL1A1, and PDGFRB) compared to HGSC suggesting that there may be more CAFs in LGSC compared to HGSC or that they have different CAF subpopulations (76).

As mentioned above, single cell sequencing data show that, in addition to the cancer cells themselves, there is a dramatic increase in immune and inflammatory cells in the tumor. This is surprising given OCs are thought to be “immunologically cold” and OC patients have only a 10-15% response rate to immune checkpoint inhibitors (174). Due to this discrepancy, studies have sought to stratify the OC TME into two categories based on the TME immune activity levels: low or high TME activity (lTME/hTME). These terms are interchangeable with having an ‘immunologically cold’ or an ‘immunologically hot’ tumor. The hTME or “hot tumor” shows higher levels of CAFs, M1 macrophages, and CD8^+^ T-cells; therefore, the TME was more active in immune infiltration, proinflammatory, and was more responsive to drug therapies. This category is more common in individuals under 65 years of age, and they typically have a better prognosis (175). The lTME or “cold tumor” has lower levels of immune cells, including B-cells, T-cells, fibroblasts, NK cells, dendritic cells, and macrophages. Although, when measuring mTOR and TGF-β signaling pathways, the lTME showed higher levels of immune suppression (154). Also, in general many of the immune cells in the metastatic sites of the omentum are immunosuppressive in nature with a high levels of pro-tumor neutrophils that help establish pro-metastatic sites as well as regulatory and exhausted T-cells, tumor associated macrophage (TAMs), and plasma cells that express pro-tumorigenic factors such as VEGF and TGF-β (173).

The immune suppression proteins, PD1 and LAG3, have been identified to be co-express in OC, which indicates high tumor immune evasion rates (175). Also, PD-L1, is highly expressed in various EOC samples; yet these levels did not influence the patient survival (176). Despite the high levels of immune checkpoint proteins expressed in OC there has been poor response of OC tumors to immune checkpoint inhibitors. This could be because of the complexity of OC immune cell microenvironment where there are immune cell compartments within the tumor, the ascites, and the omentum that all can influence the response of the tumor to the immune checkpoint inhibitors. This is shown by tumor, endothelial and mesothelial cells in metastatic sites having the ability to express high levels of NECTIN2 which interacts with TIGIT-expressing T-cells inhibiting their function and could be an alternative immune checkpoint target in OC (173). Also, the HGSC cells tend to decrease their expression of HLA proteins and the metastatic tumor sites have lower expression than the primary site (68). This suggests that the immune checkpoint inhibitors are not working in OCs due to a loss of cancer cell targeting by the T-cells which need HLA binding to function properly. This suggests that NK-cell-based therapies may represent a promising alternative approach for treatment as they target cells independent of HLA expression. Of interesting note, *post-hoc* analysis of several of the clinical trials for immune checkpoint inhibitors showed benefit for CCOC suggesting that it may have a more responsive immune microenvironment compared to other OC subtypes (177). Although the cellular and molecular reasons for these results are unclear and need further investigation.

Along with the CAFs a major driver of this immune suppression response within the tumors are macrophages. Macrophages are one of the largest cellular components of most ovarian tumors, often making up over 50% of the cells within the tumor (159, 178).

Within the ascites, macrophages are also the most prevalent immune cell, making up ~50% of all cells (179–181) and are a source of various GFs and cytokines. The omentum itself is composed of adipose cells, that have pockets within, termed “Milky spots”, from which macrophages typically enter the ascites (182, 183). These pockets contain high levels of immune and stromal cells (184). Macrophages are crucial to OC development. When they are reduced in the ascites, there is a decrease levels of vascular endothelial growth factor (VEGF) and progression of the tumor is slowed (185). Studies have shown that with patient-derived xenografts (PDX) in humanized mice that the OC cells secrete factors that recruit monocytes/macrophages to the tumors such as M-CSF, GMCSF and MCP-1 as well as cytokines that are immunosuppressive such as IL-10 (186). Further studies using organoids derived from these PDX determined that the OCs could recruit monocytes and macrophages into them with a higher rate of M2-like macrophages compared to M1 or monocytes (187). The M2-like macrophages decreased the sensitivity of the organoids to both paclitaxel and carboplatin although the mechanism of this increased resistance to treatment was not determined (187). However, changing macrophage phenotype to M1-like restores the sensitivity to treatment in both organoids and humanized mouse models (187). A better understanding of how the TME interacts with the macrophages to influence their development and how they then regulated the cells within the tumor is needed. To this end there needs to be a better understanding of TAMs heterogeneity within the different OC subtypes as well as validation of biomarkers to identify the different subpopulations of TAMs.

The importance of both TAMs and CAFs in tumor progression is highlighted by recent studies showing that one way that cancer cells survive within the ascites is forming a tri-layered spheroid with cancer cells sandwiched between the TAMs on the inside and the CAFs on the outside (68). This spheroid complex formation could be decreased using a CD44 antagonist (Angstrom6) (68). Also, tumor growth was decreased in a mouse model when treated with the CD44 antagonist or a SPP1 antibody targeting macrophages (68). These results show that targeting the interactions between the CAFs, TAMs and cancer cells is a potential area of drug targets for novel treatments. In LGSC there is a significantly lower amount of both total macrophage infiltration as well as less CD163^+^ macrophages when compared to HGSC (45). This results in a lower level of MMP9 expression and decreased micro vessel density in LGSC compared to HGSC (45). This may account for the less aggressive nature of LGSC.

T-cells respond to tumor inflammation and secreted cytokines (188); while T-cell types and levels in the OC TME are highly variable (146, 189), particularly amongst OC subtypes. T-cells have the most significant effect in the HGSC subtype (190). Survival rates increase in EOC cases with higher CD3^+^ T-cells within the tumor and in cellular clumps within the ascites; however, if these clumps are devoid of CD3^+^ T-cells there is an upregulation of VEGF expression (146). Heightened levels of CD3^+^ T-cells are also associated with better responses to chemotherapy (191). LGSC, mucinous, and CCOC have low levels of T-cells (192), which may be reasons for poorer responses to chemotherapies.

In summary, the immune-cell composition differs substantially across ovarian carcinoma histotypes. HGSC often contains abundant T-cell infiltration but is characterized by T-cell exhaustion, macrophage-rich suppression, and stromal immune exclusion. CCOC displays a spatially compartmentalized immune architecture, with plasma cells, B-cells, T-cells, macrophages, and checkpoint-ligand expression varying within the tissue. Endometrioid carcinoma shows strong molecular subtype dependence, with mismatch repair (MMR)-deficient and POLE-mutant tumors generally being more immune-rich. Mucinous carcinoma tends to have lower cytotoxic T-cell infiltration and a more stromal immune distribution, whereas LGSC remain comparatively under-characterized but show distinct immune-regulatory programs in spatial studies.

### 2.2 Extracellular matrix components

The extracellular matrix is the framework that provides structure for all organs and all solid tumors (193). ECM proteins play critical roles in cellular survival, growth, migration, and differentiation (194). The ECM is primarily composed of different types of collagens, but also includes fibronectins, laminins, glycoproteins, and proteoglycans (195, 196). As OC tumors begin to grow on the ovaries, they remodel the ECM, making it stiffer and less elastic, thereby enabling invasion and migration (197).

In HGSC the global architecture of the tumor has been shown to correlate with survival (178). ECM disorder with collagen fiber patterns that are complex and less ordered along with cellular clusters that are fragmented, and chaotic with small islands of cancer cells mixed within the stromal cells and ECM have poorer survival rates compared to patients with tumors that are well ordered with long, ordered collagen fiber patterns and large homogeneous areas of cancer cells (178). A better understanding of how these tissue architectures form within the tumor and the cells that drive it will identify new potential targets for treatment.

Specific alterations include forming thicker, shorter collagen fibers (198), increasing levels of fibronectin (199), tenascin-C (200), and tenascin-X (201). Fibronectins aid in migration, proliferation, and adhesion pathways (202, 203), and in OC cell lines (OVCAR3 and SKOV3) the addition of fibronectins increases the multicellular aggregates (204), which is the shedding of cancer cells into the ascites. Tenascin C is typically upregulated in cancers (205), which supports tumor progression through proliferation and migration pathways (206). Tenascin X, on the other hand, is typically downregulated in most cancers (207–209) but upregulated in OC (201). Tenascin X aids in the structure of collagens, manipulating and organizing the elasticity of the fibers (209). The most abundant collagen in the ECM is collagen I, which aids in resistance to chemotherapy (197). Within the basement membrane, laminins, such as LAMA5, are highly increased and correlate with poorer patient survival (210). LAMA5 plays various roles in cancer cell growth and migration and its knockdown causes metastasis to decrease (211), indicating its importance in OC. All these changes to the ECM generally increase the migratory and aggressive properties of the tumor. Spatial sequencing of treatment-sensitive and treatment-resistant patients shows potential differences within the ECM binding within the two groups with good responders having more receptors pairing for collagen 1, collagen 3 and fibronectin 1, where poor responders had collagen 18 and laminin 5 (212). Also, the receptors used were different. For example, integrin expression in the good responders seems to be more ITGA3, ITGB1, and ITGB8, where the poor responders had ITGA3, ITGA5, ITGA9, ITGAV, ITGB1, ITGB2, ITGB6, and ITGB8 (212). There is a knowledge gap in how these ECM interactions drive differences with treatment responses which only can be solved by better and more complex 3D culture systems that better mimic the TME.

### 2.3 The secreted components

The TME contains numerous secreted factors derived from the blood, CAFs, cancer cells, and immune cells. This includes stress hormones, sex hormones, GFs, cytokines (interleukins (IL), and chemokines), proteases, and metabolites. Together, these components facilitate signaling pathways that augment tumor growth, cellular survival, and metastasis. Among these, hormones, and GFs are central regulators of tumor progression.

Hormones and GFs regulate tumor proliferation and development. Stress hormones interact with the tumor via the blood (213), and the peritoneal fluid (214). Sex hormones such as estrogen, testosterone, and progesterone originate from the ovaries (215, 216), and growth factors come from immune cells within the TME and the tumor itself (185, 216). Elevated concentrations of cortisol, epinephrine, and norepinephrine and their metabolites (normetanephrine and metanephrine) have been detected in the ascites of OC patients and correlate with increased levels of inflammatory cytokines and proteins (214). These elevations may also be due to the patients depleted emotional and physical status associated with advanced disease (217–219). Increased levels of cortisol, epinephrine, and norepinephrine have been studied in various animal models; however, the direct impact on the tumor or the immune system has not been identified. Sex hormones are produced in the ovaries (220), and OC is thought of as a sex hormone-responsive cancer (221). Estrogen can be a driver of OC across all subtypes (222), and limited case studies indicate that some ovarian tumors can produce estrogens directly (223). This is rare, and cases have mainly been CCOC or endometrioid carcinoma, although this process can occur in other OC subtypes, such as HGSC (223). The amount of estrogen receptor α (224, 225) is increased in OC, promoting cellular growth, metastasis, and angiogenesis (221, 225–227). Testosterone is rarely overproduced in OC cases, yet it is elevated in sex cord-stromal OC and, in rare cases, in endometrioid carcinomas, resulting in higher androgen levels in these cases (228). Progesterone is a protective hormone (229); however, the levels in OC are not well understood (230). In LGSC, some lack progesterone receptors, and these patients have reduced overall survival (231), suggesting the potential importance of this receptor.

Growth factors are produced by a wide variety of cells within the tumor (cancer cells, fibroblasts, immune cells) and surrounding tissues such as the omentum (adipose cells, fibroblasts, mesothelial cells), that typically aid in the spread and growth of cancer (185). VEGF levels increase with the progression of all OC subtypes, leading to poorer patient survival, and after surgical removal of the tumor, these levels significantly decrease (232). This increase is associated with reduced levels of T-cells within the ascites, aiding tumor cells in avoiding immune surveillance (233). Clinical trials of VEGF signaling inhibitors have shown that they are modestly helpful in treating patients and have been approved for clinical use. However, biomarkers capable of identifying the patients most likely to benefit from VEGF inhibition remain poorly defined. Also, other targets in angiogenesis related pathway need to be explored. For example, endothelin-1 (ET-1) has also been found to be upregulated in HGSC (234). The ET-1 receptors are expressed on the cancer cells as well as fibroblasts, macrophages and endothelial cells and drives a feed-forward loop with VEGF receptor signaling (234). Macitentan is a clinically approved inhibitor of the ET-1 receptors and represents a potential therapeutic candidate for HGSC, either alone or in combination with VEGF inhibitors (234). Epithelial growth factor (EGF) and transforming growth factor β (TGFβ) promote cancer cell migration to secondary sites via EMT in OC cases (235). These growth factors aid in the initial movement from the fallopian tubes to the ovaries, then from the ovaries to the peritoneal surfaces, etc. EGF activates EMT, and TGFβ further increases activity levels while reducing epithelial marker levels (235). Indicating that GFs are essential for both the growth and aggressiveness of the tumors, but also the increased migratory factors in this type of cancer.

Cytokines are small proteins that act as chemical messengers for immune and inflammatory cells; this group covers interleukins, interferons, and chemokines (236, 237). In OC, cytokines that have been studied for their roles in the TME include IL-4, -6, -8, -10, -12, TNF-α, TGF-β, and other chemokines. IL-4 is increased in the ascites of the OC tumor (238), and the receptor for IL-4 can interact with PD-1 which can lead to anti-PD-1 treatments failing to alter tumor growth (239). TNF-α contributes to general inflammation, angiogenesis, and metastasis, which are upregulated in OC (240–242). IL-6 is produced in response to the increased levels of proinflammatory cytokines (243) and is observed at levels around six times higher in ascites of OC patients than in healthy individuals (244, 245). Within ascites, IL-6 promotes neutrophil recruitment, and, together with TGF-β drives CD4^+^ T-cells differentiation and IL-21 production (246–248). IL-6 also enhances VEGF production (249) while reducing proteins involved in wound healing (250), potentially contributing to poor prognosis (232). The increase in neutrophils also upregulates IL-8 production (251), which also promotes tumor growth and migration (252). Unlike the previous cytokines, IL-10 is generally believed to be anti-inflammatory (253),; and yet, it is increased in OC (254). In OC, IL-10 seems to be pro-tumorigenic, having roles in increasing tumor growth (255), cell migration specifically in later stages (256) and increasing the immunosuppressive environment of OC (257). In OC, IL-12 is cytotoxic due to recruiting various immune cells, like natural killer cells, to kill cancerous cells (258, 259); therefore, high levels of IL-12 are associated with higher OC patient survival (260). Studies on various cytokines and their effects on OC subtypes could lead to a better understanding of OC TME and their responses to treatment.

By preserving tissue architecture, spatial sequencing approaches can identify neighboring cell populations and infer possible autocrine and paracrine signaling pathways through ligand–receptor co–expression (212, 261, 262). These include ligand-receptor pairs, such as GPC3-IGF1R and GPC3-CD81, that have not previously been identified in the HGSC subtype (212). GPC3 is known to be overexpressed in CCOC (263) but not HGSC where these interactions were found. Other studies have shown ligand-receptor pairs that would indicate communication between the cancer cells and TAMs and/or CAFs (261). Many of these ligand-receptor pairs are not well characterized in OC and represent new targets for drug research.

Although numerous proteases are overexpressed in OC, only a few have been shown to affect patient survival. Matrix metalloproteinases (MMPs) 2 and 11 are both increased when the cancer spreads to the peritoneum, but the MMP2 is the only one shown to negatively affect overall survival (264). MMP1, 3, 7, 9, and 14 have also been shown to be upregulated in OC cases (265–268). MMP1, 3, and 7 were specifically shown to be expressed in the malignant cell lines OVCAR3 and SKOV3 (269) and were found to cause ECM collagen breakdown in studies (270, 271). MMP7 is also associated with poor patient survival (269). Various kallikreins (KLK), a family of serine proteases, are upregulated in OC (272) and are shown to drive cell proliferation, angiogenesis, and cellular migration (273). *ADAMTS* influences cellular migration, invasion, and metastasis; in OC, it is upregulated, leading to reduced overall patient survival (274). Missense mutations in *ADAMTS* are common; they are associated with improved patient survival and better responses to chemotherapy or platinum-based therapies (275). Many proteases are differentially expressed in OC; in spite of that, limited research has examined the effects of these upregulations on the tumor and the TME.

Ovarian cancers also remodel the TME by altering metabolites to create a favorable environment for tumor growth. Different levels of amino acids have been identified in OC; however, the causes of these changes remain unknown. Citrulline is significantly reduced in OC tumors (276). Citrulline has been shown to trigger a stronger immune response (277); therefore, a reduction may lessen the immune response. Aminobutyric acid, sarcosine, and glutamic acid are also reduced in OC compared to normal tissues, and disturbances are seen in various amino acid-related pathways. These pathways including arginine biosynthesis and the metabolism of butanoate, alanine, aspartate, glutamate, glycine, serine, threonine, arginine, and proline although the role of these alterations in OC is not clear. Lysophosphatidic acids (LPAs) are increased (278, 279), thereby aiding the production of GFs, MMPs, and cytokines (279). The omentum is rich in adipocytes (280), which release fatty acids into the ovarian TME (281). OC cells overexpress fatty acid synthase, causing fatty acid synthesis to be highly active (282). Fatty acids can then interact with phosphatidylinositol, for example, increasing interactions with tyrosine kinases (283), thereby activating the PI3K-mTOR pathway (284). They also promote the growth of cancerous cells by increasing fatty acid uptake in cells via the receptor CD36, which the cancer cells express (285). To produce additional energy (adenosine triphosphate), these fatty acids are oxidized, which can then aid cancer cell growth.

## 3 Discussion

**Figure 4:**
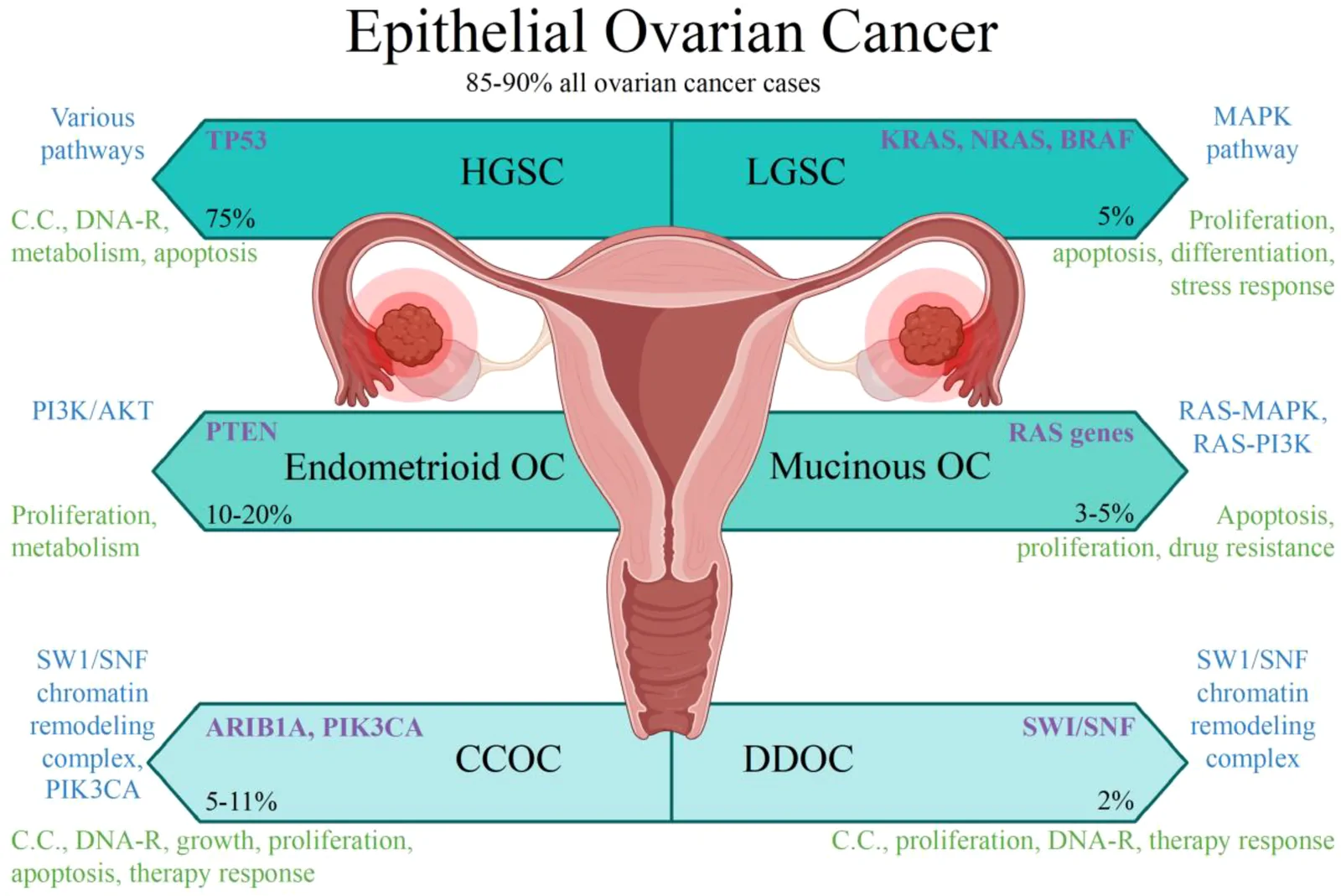
The complexity of epithelial ovarian cancer subtypes. Each subtype shows the percentage of epithelial ovarian cancer cases and the mutation frequency in those cases (purple). Then, in blue indicates the pathways or complexes affected by the frequent mutation. Moreover, in green are the cellular responses caused by the alterations in pathways. HGSC, high-grade serous ovarian cancer. LGSC, low-grade serous ovarian cancer, CCOC, clear cell ovarian carcinomas, and DDOC, dedifferentiated ovarian carcinomas. C.C., cell cycle. DNA-R, DNA-repair.

Ovarian cancer is a complex disease (**Figure 4**) characterized by dynamic interactions among the cells of the tumor, ascites, and omentum. Although numerous studies have examined the individual components, they have often done so in isolation, overlooking the bidirectional and therapeutic response. For instance, immune cells within OC TME may originate from either blood vessels within the ovary or the “milky spots” in the omentum. Likewise, the omentum produces fatty acids and amino acids as a major energy source for the support of tumor development. To fully understand the development of the OC tumor, requires an integrated view of the interactions between the cellular, structural, and secreted components of the TME. Studying these compartments independently risks overlooking critical mechanisms of intercellular communication and metabolic crosstalk that influence disease progression.

There are several types and subtypes of OC, with EOC being the most common and HGSC being the most prevalent subtype. While other EOC subtypes exist, they are significantly less common and are thus researched less extensively. Examples of such include LGSC, endometrioid, mucinous, CCOC, and DDOC. Additionally, germline, stromal cells, and borderline OC types are also under-researched. The tumors and TME associated with these various types and subtypes behave distinctly from those of HGSC. This means that the interactions and roles of the three TME components (tumor, ascites and omentum) of OC may vary significantly. As a result, biomarkers and treatment strategies developed primarily from HGSC studies may not translate effectively to other OC subtypes, potentially limiting treatment efficacy and contributing to disease recurrence.

Future studies should therefore adopt integrated approaches that examine the structural, cellular, and molecular interactions across the entire OC TME while incorporating the biological diversity of OC subtypes. Advances in spatial sequencing, single-cell technologies, and physiologically relevant three-dimensional culture models provide an opportunity to better define these complex interactions. A more comprehensive understanding of the OC TME will facilitate the identification of clinically actionable therapeutic targets and biomarkers, ultimately enabling more personalized treatment strategies and improving patient outcomes.
